# The Role of Phospholipase D in Modulating the MTOR Signaling Pathway in Polycystic Kidney Disease

**DOI:** 10.1371/journal.pone.0073173

**Published:** 2013-08-23

**Authors:** Yang Liu, Andres Käch, Urs Ziegler, Albert C. M. Ong, Darren P. Wallace, Alexandre Arcaro, Andreas L. Serra

**Affiliations:** 1 Institute of Physiology, University of Zurich, Zürich, Switzerland; 2 Center for Microscopy and Image Analysis, University of Zurich, Zürich, Switzerland; 3 Academic Unit of Nephrology, Department of Infection and Immunity, University of Sheffield, Sheffield, United Kingdom; 4 Department of Medicine, University of Kansas Medical Center, Kansas City, Kansas, United States of America; 5 Department of Clinical Research, University of Bern, Bern, Switzerland; University of Florida, United States of America

## Abstract

The mammalian target of rapamycin (mTOR) signaling pathway is aberrantly activated in polycystic kidney disease (PKD). Emerging evidence suggests that phospholipase D (PLD) and its product phosphatidic acid (PA) regulate mTOR activity. In this study, we assessed *in vitro* the regulatory function of PLD and PA on the mTOR signaling pathway in PKD. We found that the basal level of PLD activity was elevated in PKD cells. Targeting PLD by small molecule inhibitors reduced cell proliferation and blocked mTOR signaling, whereas exogenous PA stimulated mTOR signaling and abolished the inhibitory effect of PLD on PKD cell proliferation. We also show that blocking PLD activity enhanced the sensitivity of PKD cells to rapamycin and that combining PLD inhibitors and rapamycin synergistically inhibited PKD cell proliferation. Furthermore, we demonstrate that targeting mTOR did not induce autophagy, whereas targeting PLD induced autophagosome formation. Taken together, our findings suggest that deregulated mTOR pathway activation is mediated partly by increased PLD signaling in PKD cells. Targeting PLD isoforms with pharmacological inhibitors may represent a new therapeutic strategy in PKD.

## Introduction

Autosomal dominant polycystic kidney disease (ADPKD) is a inherited kidney disease characterized by progressive development of fluid-filled cysts in both kidneys, which results in end-stage renal disease in approximately 50% of affected individuals by the sixth decade of life. ADPKD is caused by mutations in the *PKD1* (approximately 85%) and *PKD2* (approximately 15%) genes encoding polycystin-1 and 2 (PC1 and PC2). PC1 and PC2 function in cell-cell and cell-matrix interactions, signal transduction and mechanosensation [1,2]. A direct physical interaction exists between the cytoplasmic tail of PC1 and the tumor suppressor tuberin, the product of the TSC2 gene that regulates the kinase activity of mTOR. Mutations in PC1 disrupt this interaction, unleashing mTOR and in turn, promote the proliferation of cyst-lining epithelial cells in ADPKD by aberrant signaling through mTOR [3]. . 

mTOR is a Ser/Thr kinase that governs a wide variety of biological and cellular processes, including cell growth, proliferation, survival and autophagy [4,5]. mTOR is composed of two functionally and structurally distinct complexes: mTORC1 and mTORC2 [6]. The binding of raptor to mTOR defines the nutrient-sensitive mTORC1 that regulates protein synthesis by phosphorylating its substrates the 4E-binding protein1 (4E-BP1) and the 70-kD ribosomal S6 kinases (S6K) [7]. Rapamycin in a complex with its intracellular receptor FKBP12 specifically binds to the FKBP12/rapamycin binding domain of mTOR and inhibits mTORC1 function. mTORC2, assembled by the binding of rictor, a rapamycin-insensitive companion of mTOR, is activated by growth factors alone. The commonly described substrate of mTORC2 is Akt at the Ser473 site [8].

Phosphatidic acid (PA), a phospholipase D (PLD) product generated by the hydrolysis of phosphatidylcholine, regulates mTOR activity [9]. PLD is activated by a variety of hormones, growth factors and cytokines. Two PLD isoforms are expressed in most mammalian tissues: PLD1 and PLD2, which are endowed with different properties, regulatory mechanisms and functions [10]. PA is required for the stability of mTORC1 and mTORC2 and modulates the kinase activity of both complexes. PA interacts with mTOR in a manner that is competitive with rapamycin. As a consequence, elevated PLD activity confers rapamycin resistance [11]. Aberrant PLD/PA signaling has been observed in a number of human carcinomas, including breast, ovary, kidney and colon cancer [12–14]. The elevated PLD activity in human carcinomas is thought to promote cell proliferation and to suppress the default apoptotic programs, thereby promoting cancer growth. We hypothesized that PLD activity governs PKD associated cell proliferation via the mTOR signaling pathway in PKD; however this has not been examined yet.

Autophagy, also called “self-eating”, is an evolutionarily conserved cellular pathway whereby cytosolic components are targeted for removal into membrane-bound compartments, named autophagosomes [15]. Autophagy has been well established as a cytoprotective mechanism under stress conditions, such as starvation. A number of studies have provided evidence that inadequate levels of autophagy can also lead to non-apoptotic cell death [15,16]. As mTOR signaling modulates autophagy and abnormally increased mTOR signaling is a feature of PKD, a connection between autophagy and PKD has been proposed [17]. However, there is so far only one report showing abnormalities in autophagy and autophagy-related proteins in PKD animal models [18].

In the current study, we show for the first time that PLD activity is abnormally elevated, and partly contributes to mTOR pathway activation in PKD cells. The mTOR signaling pathway is modulated in a PLD-dependent way in PKD. Inhibition of PLD activity increased the inhibitory effect of rapamycin on mTOR. Furthermore, targeting PLD impaired cell proliferation and induced autophagy, which may represent an opportunity for the development of new treatment strategies for PKD [19].

## Materials and Methods

### Antibodies and reagents

All commercial antibodies and chemicals were purchased from the following suppliers: anti-phospho-(T308)-Akt (4056), anti-phospho-(S473)-Akt (4051), anti-phospho-(T389)-p70 S6K (9206), anti-phospho-(T421/S424)-p70 S6K (9204), anti-p70 S6K (2708), anti-LC3B (3868), anti-caspase 3 (9662), anti-phospho-(S561)-PLD1(3834), anti-PLD1(3832), anti-phospho-(S235/236)-S6 (2211), anti-S6, anti-phospho-(T37/46)-4EBP1 (9459), anti-4EBP1(9644), anti-phospho-(S2448)-mTOR (2971), anti-mTOR (2983), anti-Atg5 (8540) antibodies were from Cell Signaling Technology; anti-Akt (ab8805-200) was from Abcam; anti-GAPDH (MAB374) was from Merck Millipore; anti-phospho-(Y169)-PLD2 (A8400) was from Assay Biotech; anti-PLD2 (sc18532) was from Santa Cruz; Sheep anti-mouse IgG-HRP (NA931A) and donkey anti-rabbit IgG-HRP (NA934A) were from GE Healthcare; Alexa Fluor 488 donkey anti-rabbit IgG (A21206) was from Life technology; 1,2-dioleoyl-*sn*-glycero-3-phosphoric acid monosodium salt (DOPA, 74304), rapamycin (R0395), honokiol (H4914), 1-BtOH (B7906) and *tert*-BtOH (19460) were from Sigma-Aldrich AG. PLD1 and PLD2 inhibitors were kindly provided from Prof. H. Alex Brown and Prof. Craig W. Lindsley.

### Primary and immortalized renal tubular epithelial cell cultures

The Han: SPRD rat colony was established in our animal facility from a litter which was obtained from the Rat Resource & Research Center (Columbia, MO, USA) and kept under local regulation and guidelines. The animal study was approved by the animal health regulatory agency of the Canton Zürich, Switzerland. Heterozygous cystic (Cy/+) and wild-type normal (+/+) male rats, aged 8-weeks-old were used in this study. Primary renal epithelial cells were isolated as follows: kidneys were minced and digested by 1 mg/ml collagenase with gentle agitation for 1 hour at 37°C. The suspension was allowed to sediment for 1 minute twice. Cells were collected by harvesting the supernatant and then washed 3 times with 10% FBS/HBSS. Isolated cells were re-suspended in K1 medium (1:1 mixture of Dulbecco’s modified Eagle’s medium and Ham’s F-12 medium supplemented with 5% FBS,10 mM HEPES, 42 mM sodium bicarbonate, 50 ng/ml insulin, 50 nM hydrocortisone, 50 ng/ml transferrin, 5 pM trijodothyronine, 100 IU/ml penicillin, and 100 µg/ml streptomycin). Cells were seeded in collagen type 1-precoated culture dishes.

Primary cultures of normal human kidney epithelial cells (NHK) and ADPKD cyst-lining renal epithelial cells (ADPKD) were generated by the PKD Research Biomaterials and Cellular Models Core at the University of Kansas Medical Center (Kansas City, KS, USA) [20]. Human immortalized cystic (OX161) and noncystic (UCL93) cells were kindly provided by Prof. A.C. Ong (University of Sheffield, Sheffield, UK) [21]. The culture conditions of human primary and immortalized renal epithelial cells were the same as for rat primary renal tubular epithelial cells.

### Cell viability and proliferation assays

The 3-(4,5-dimethylthiazol-2-yl)-5-(3-carboxymethoxyphenyl)-2-(4-sulfophenyl)-2H-tetrazolium (MTS)-based CellTiter 96 AQueous One assay (G3581, Promega) was used to quantify cell viability. Cell proliferation was assessed using BrdU Cell Proliferation ELISA Kit (11647229001, Roche Applied Science), which quantifies cell proliferation by measuring DNA synthesis. The assay was performed according to the manufacturer’s instructions and results were expressed as mean absorbance of the samples measured in an ELISA plate reader.

### Western blot analysis

Total cell lysates were prepared in the ice-cold lysis buffer containing 40 mM Hepes, 120 mM NaCl, 1 mM EDTA and 1% Triton (pH 7.5), supplemented with proteinase inhibitors (Roche) and phosphatase inhibitors (10 mM potassium pyrophosphate, 10 mM sodium β-glycerophosphate, 50 mM sodium fluoride, 0.5 mM sodium orthovanadate). The amount of protein was determined by using the BCA Protein Assay (23225, Thermo Scientific). Cell lysates in SDS-sample buffer were boiled for 10 minutes at 95^º^C and equal protein amounts were resolved by SDS-PAGE, immunoblotted using polyvinylidene difluoride membranes and probed with antibodies. Signals were visualized by using the Chemiglow West chemiluminescence substrate kit (60-12596-00, ProteinSimple).

### Preparation of PA

DOPA (74304, Sigma-Aldrich AG) was dissolved in chloroform and dried under nitrogen. The lipid film was resuspended in vesicle buffer (150 mM NaCl and 10 mM Tris-Cl (pH8.0)) by vortexing briefly to yield a final lipid concentration of 10 mM. The lipid suspension was then sonicated in a water bath sonicator for 5 min. This procedure is expected to yield small unilamellar vesicles with diameters in the range of 15-50 nm. Lipid vesicles were made freshly before each experiment and were added directly to cell medium at the final concentration of 100 µM. Due to the short half-life of PA, this process was repeated every 40 min throughout treatment.

### PLD enzyme activity

The protein samples were collected and the concentration determined using the same procedures as describes for the Western blot assay. 50 µg of protein samples were used to determine PLD activity with the Amplex Red PLD assay kit (A12219, Invitrogen), according to the manufacturer’s protocol.

### Immunofluorescence staining

Cells were seeded into 6-well plates with coverslips. 24 h later, cells were treated with or without PLD inhibitors for 48 h. Cells grown on the coverslips were fixed and permeabilized with ice-cold methanol at -20°C for 10 min. After 3 washes with PBS, the samples were blocked with blocking buffer (1% BSA, 1% Triton X-100 in PBS (pH 7.4)) at room temperature for 30 min. The samples were then incubated with primary antibody (anti-LC3B) at room temperature for 2 h. After 3 washes with PBS, the samples were incubated with Alexa Fluor 488 donkey anti-rabbit IgG at room temperature avoiding light for 1 h. Slides were examined by using a laser scanning confocal microscope (Leica SP5).

### Transmission electron microscopy

ADPKD and OX161 cells treated with or without either 10 µM PLD1 inhibitor or 20 µM PLD2 inhibitor for 48 h were fixed for 2 h at room temperature (RT) with 2.5% glutaraldehyde in PBS (pH 7.4) and, subsequently, with 1% OsO4 in 50 mM sodium cacodylate buffer (pH 7.3), dehydrated in an ethanol series and embedded into epon (Catalyst). Ultrathin sections of 50 nm were contrasted with uranyl acetate and lead citrate and analyzed in a Tecnai Spirit transmission electron microscope (FEI) with an ORIUS CCD camera (Gatan).

### Statistical analyses

Statistical analyses were performed by one-way ANOVA with the Dunnet post-hoc test. All data are expressed as means ± SD. P values were two sided for the comparison between the groups or between baseline and follow-up values, and those less than 0.05 were considered statistically significant.

## Results

### Elevated mTOR and PLD activity in PKD cells

**Figure 1:**
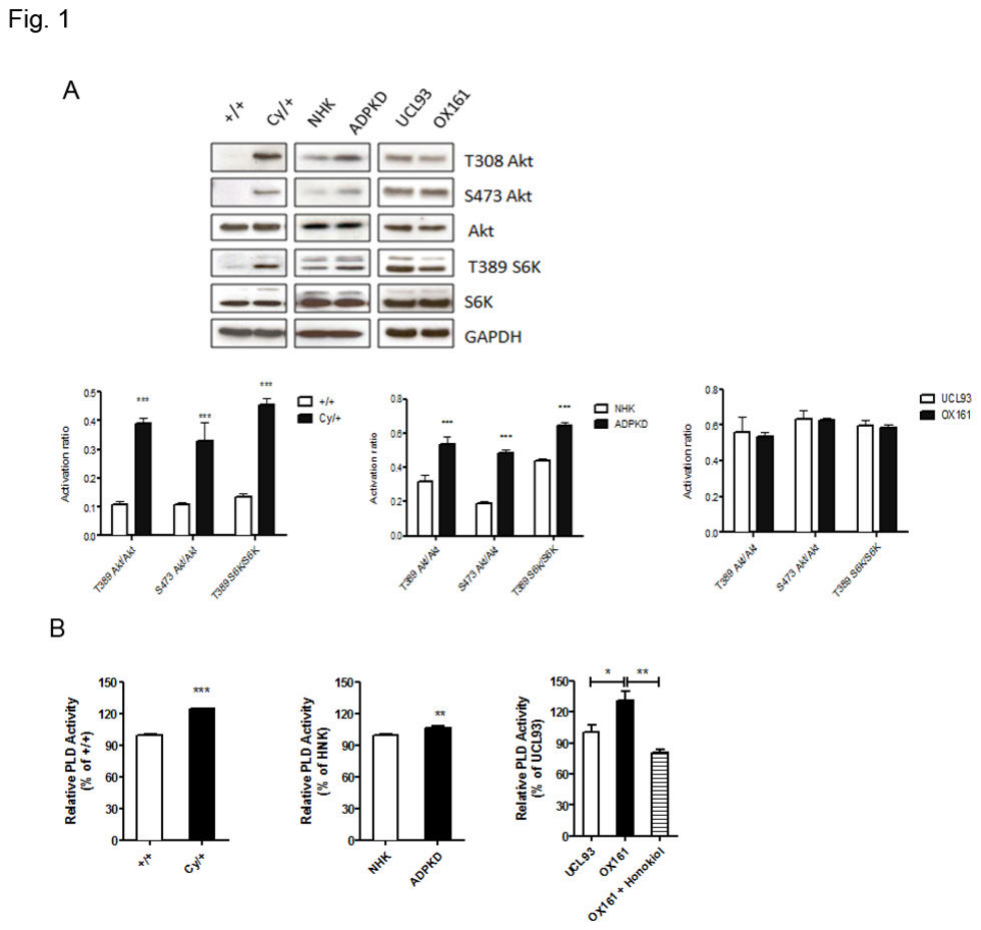
Elevated mTOR and PLD activity in PKD cells. (A) Western blots and densitometry analyzing the endogenous activity of the mTOR in PKD and control cells. (B) The endogenous level of PLD activity was determined in PKD and control cells by the Amplex Red PLD assay kit. Honokiol (20 µM) treated OX161 for 20 h as a positive control. An experiment which is representative of three independent experiments is shown. Data are expressed as mean ± SD and were analyzed by one-way ANOVA. * p< 0.05, ** p< 0.01, *** p< 0.001.

In this study, we used 3 different PKD cellular models: 1) primary renal tubular epithelial cells derived from heterozygous Cy/+ and +/+ Han: SPRD rats, a well characterized strain (Cy/+), which phenotypically resembles human ADPKD [22]; 2) an immortalized human ADPKD renal tubular epithelial cell line (OX161) and an immortalized normal renal tubular epithelial cell line (UCL93) [21]; and 3) primary renal epithelial cells derived from ADPKD patients (ADPKD) and from normal human kidney tissue (NHK) [23]. Western blots analysis was used to examine the expression of regulators and effectors of mTOR complexes in the PKD renal cystic epithelial cells, and were compared with normal renal tubular epithelial cells. Figure **1A** shows the markers of activation of mTORC1 (phosphorylation at T308 of Akt, and at T389 of S6K) and mTORC2 signaling pathway (phosphorylation at S473 of Akt). Mutations in *PKD1* or *PKD2* lead to profound effects on downstream target tuberous sclerosis complex (TSC)-mTOR pathway in ADPKD. In our study, the basal activity of mTOR, assessed by the phosphorylation status of the mTOR readouts Akt and S6K, was higher in the primary PKD cells, compared with normal renal epithelial cells. In the immortalized cells, we observed that both OX161 and UCL93 cells had high activation of Akt and S6K, which suggests that the elevated mTOR pathway activity is associated with increased cell proliferation in immortalized PKD and normal cells.

Since it was recently shown that aberrant PLD activity is associated with several human cancers, and PA, a product of PLD activation, is required for the stability and activity of the mTOR complex, we hypothesized that the abnormal mTOR pathway activation could be partly due to high basal PLD activity in PKD. To test this hypothesis, we examined endogenous PLD activity by measuring its activity *in vitro*. In this study, we used honokiol to treat OX161 as a positive control when measuring PLD activity with the specific enzymatic assay [24]. Honokiol, a bioactive compound obtained from several species of the genus *Magnolia* of Magnoliaceae family, suppressed the PLD activity through targeting the activation of the upstream regulator Ras [25]. The data presented in Figure **1B** shows that the basal level of PLD activity is increased in all investigated PKD cell models compared with normal renal epithelial cells and that honokiol decreased PLD activity of OX161 cells.

### PLD Inhibitors Impair Cell Proliferation in Renal Tubular Epithelial Cells

**Figure 2:**
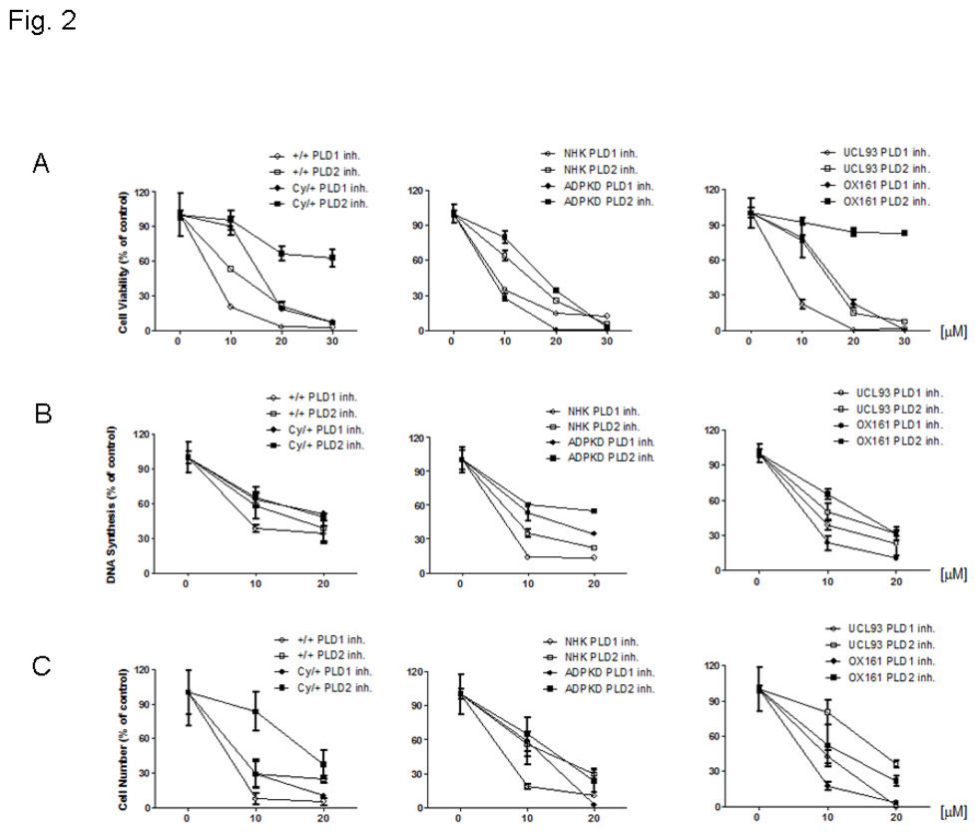
PLD inhibitors impair cell growth and block PLD activity in PKD cells. Effect of PLD1-/2-inhibitors on (A) the cell viability, (B) DNA synthesis ability and (C) cell proliferation were determined by MTS, BrdU and cell counting, respectively. All types of cells were exposed to various concentrations of either PLD1 or PLD2 inhibitor for 48 h. An experiment which is representative of three independent experiments is shown.

Since up-regulated PLD activity was observed in all PKD cells we hypothesized that targeting PLD could be an effective treatment strategy for PKD. We therefore examined the efficacy of specific inhibitors of PLD1 and PLD2 on PKD cell proliferation which was determined by MTS, BrdU and cell counting assays [26]. The PLD inhibitors we used as Scott SA et al. reported that directly interact with the catalytic domains of the enzyme, and do not require binding with the regulatory PX-PH domain to execute inhibition [26]. Figure **2** shows that both PLD1 and 2 inhibitors reduced cell viability, DNA synthesis and cell proliferation in normal and PKD cells in a dose-dependent way. Both PLD inhibitors proved cytotoxic in all cell types and the PLD1 inhibitor exhibited a higher efficacy in reducing cell proliferation compared with the PLD2 inhibitor.

### PLD inhibitors specific target PLD

**Figure 3:**
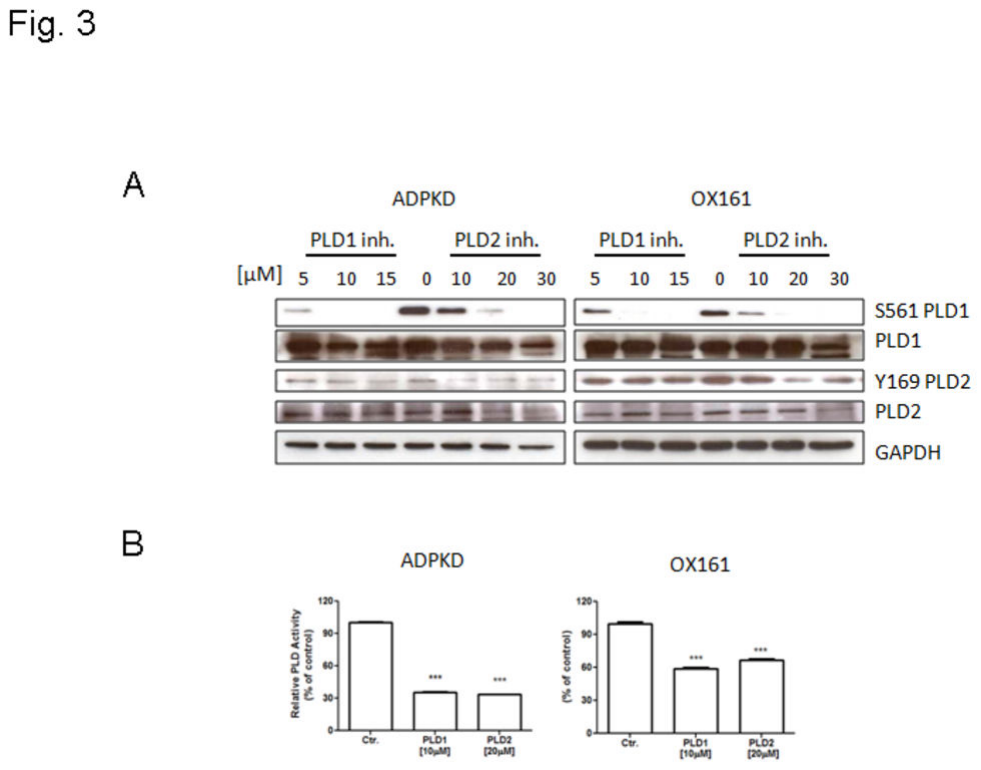
PLD inhibitors block the PLD/PA pathway. (A) Western blots analyzing the expression of phospho-PLD1, PLD1, phospho-PLD2, PLD2 and GAPDH either upon treatment with different concentrations of PLD1-/2- inhibitor (for PLD1 inhibitor: 5, 10, 15 µM, for PLD2 inhibitor: 10, 20, 30 µM) treatment for 48 h or without treatment. (B) PLD enzyme activity assay determining PLD activity upon treatment with either 10 µM PLD1 inhibitor or 20 µM PLD2 inhibitor for 48 h. An experiment which is representative of three independent experiments is shown. Data are expressed as mean ± SD and were analyzed by one-way ANOVA. ***p < 0.001.

We next focused our study on human PKD cells to examine whether PLD inhibitors could specifically block PLD phosphorylation. Western blot analysis indicated that the dose-dependent effect of PLD inhibitors with the reduced phosphorylation levels of PLD1 and PLD2. Figure **3A** shows that both PLD1 and PLD2 inhibitors reduced specifically the phosphorylation of PLD1 and PLD2, respectively. Using an enzymatic assay to correlate the inhibition of phosphorylation with PLD activity we found that both PLD inhibitors reduced total PLD activity in ADPKD and OX161 cell lysates (Figure **3B**). Inhibition of PLD activity was dose-dependent and maximal at doses of 10 µM and 20 µM for the PLD1 and PLD2 inhibitor, respectively.

### PLD inhibitors affect the mTOR signaling pathway, while exogenous PA stimulates mTOR signaling

**Figure 4:**
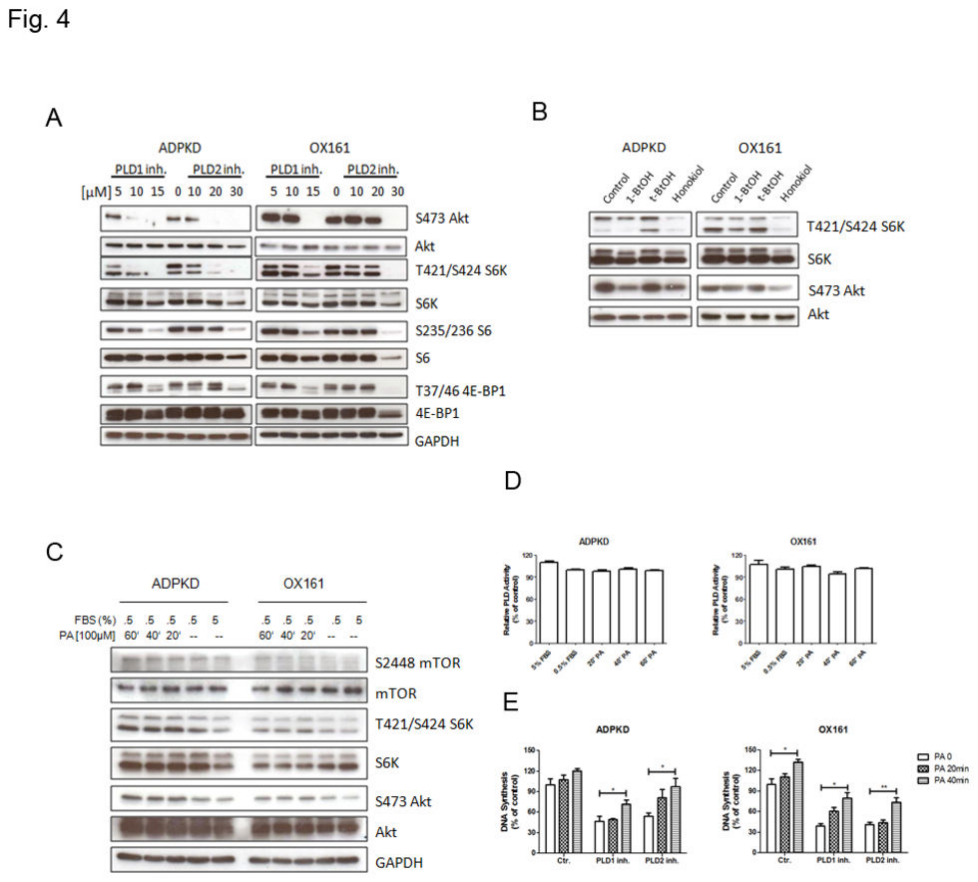
PLD inhibitors block mTOR signaling pathway and exogenous PA abrogates the effect of PLD inhibitors on mTOR signaling and proliferation of PKD cells. (A) PLD inhibitors affect mTOR signaling in a dose-dependent way. Western blots analyzing the expression of phospho-Akt, Akt, phospho-S6K, S6K, phospho-S6, S6, phospho-4EBP1, 4EBP1 and GAPDH either upon treatment with the indicated concentration of PLD1-/2- inhibitor for 48 h or without treatment. (B) PKD cells were plated for 24 h and then shifted to medium containing 0.5% serum. 1% 1-BtOH or 1% *tert*-BtOH was added for 2 h, 20 µM honokiol was added for 20 h. Western blots analyzing the expression of phospho-S6K, S6K, phospho-Akt and Akt. (C, D) Exogenous PA stimulated mTOR signaling in a time-dependent way, but not PLD activity. Human PKD cells were cultured under either normal culture medium (5% FBS) or starvation culture medium (0.5% FBS). After 24 h starvation, cells were treated with 100 µM PA for 20, 40 and 60 minutes. Whole cell lysates were analyzed by (C) western blots to detect the expression of phospho-mTOR, mTOR, phospho-S6K, S6K, phospho-Akt, Akt and GAPDH. (C) Whole cell lysates were determined by Amplex Red PLD kit to measure the PLD activity. (E) Exogenous PA impairs the effect of PLD inhibitors on cell growth. Cells were pretreated with either 10 µM PLD1 inhibitor or 20 µM PLD2 inhibitor for 48 h, and then with 100 µM PA for the indicated time. DNA synthesis was measured by using the BrdU assay. An experiment which is representative of three independent experiments is shown. Data are expressed as mean ± SD and were analyzed by one-way ANOVA. * p< 0.05, ** p< 0.01.

PA is known to mediate the activation of mTOR signaling by binding to the FKBP12-rapamycin binding (FRB) domain of mTOR. Since we found that the activity of PLD was elevated in PKD, we tested the hypothesis that PLD activity is required to sustain the activation the mTOR signaling pathway in human PKD cells. Thus we treated ADPKD and OX161 cells with PLD1 and PLD2 inhibitors and examined their effect on the phosphorylation of the up- and down-stream targets of the mTOR pathway. We found that the phosphorylation of both the up-stream targets of mTORC1 (phospho-T308-Akt) and the down-stream targets (phospho-T421/S424-S6K, phospho-S235/236 S6, phospho-T37/46-4E-BP1) decreased in a dose-dependent manner (Figure **4A**). The readout for mTORC2, phospho-S473-Akt, also decreased upon PLD1 and PLD2 inhibitor treatment (Figure **4A**). To further assess the specificity of PLD inhibitors to block the mTOR pathway, we performed the “alcohol trap” assay. This alternative method of inhibiting PLD activity takes advantage of the fact, that PLD preferentially utilizes primary alcohols (1-BtOH) in the transphosphatidylation reaction, producing phosphatidyl alcohols instead of PA; whereas tertiary alcohols (*tert*-BtOH) is not a substrate for PLD and is therefore used as a negative control [27]. As shown in Figure 4B, 1% 1-BtOH suppressed the phosphorylation T421/S424-S6K and S473-Akt, whereas 1% tert-BtOH had no effect on the phosphorylation status, indicating that the observed, effects of PLD 1 and 2 inhibitors on the mTOR pathway were due to the suppression of PA production by PLD. As a second alternative method to block PLD activity, we used honokiol, considered as PLD1 and PLD2 inhibitor, to treat PKD cells. Figure 4B and Figure S1 shows, that also honokiol suppressed the phosphorylation of S6K and Akt in human ADPKD and OX161 cells. Taken together, our studies indicate that PLD 1 and PLD 2 inhibitors suppress mTOR signaling in PKD cells by decreasing specifically PLD activity.

To study the functional relevance of the inhibitory effect of PLD inhibitors on PLD activity, we examined the effect of bypassing the need for PLD activity on both mTOR signaling and cellular proliferation by adding exogenous PA to the cell culture medium. Because exogenous PA has a short half-life, we analyzed three short time points (20 min, 40 min and 60 min) of treatment with PA in both ADPKD and OX161 cells. We found that exogenous PA (100 µM) was able to stimulate by itself the activation of mTOR (S2448), S6K (T421/S424), Akt (S473 and T308) (Figure **4C**) without affecting PLD activity (Figure **4D**). Importantly, exogenous PA increased DNA synthesis in human PKD cells, and rescued the inhibitory effect of PLD inhibitors in a time-dependent manner (Figure **4E**). These results suggest that the mechanism of action of PLD inhibitors on cellular proliferation is via inhibition of PLD-dependent PA production and is partly mediated through an inhibition of the mTOR signaling pathway.

### PLD inhibitor triggers autophagosome formation

**Figure 5:**
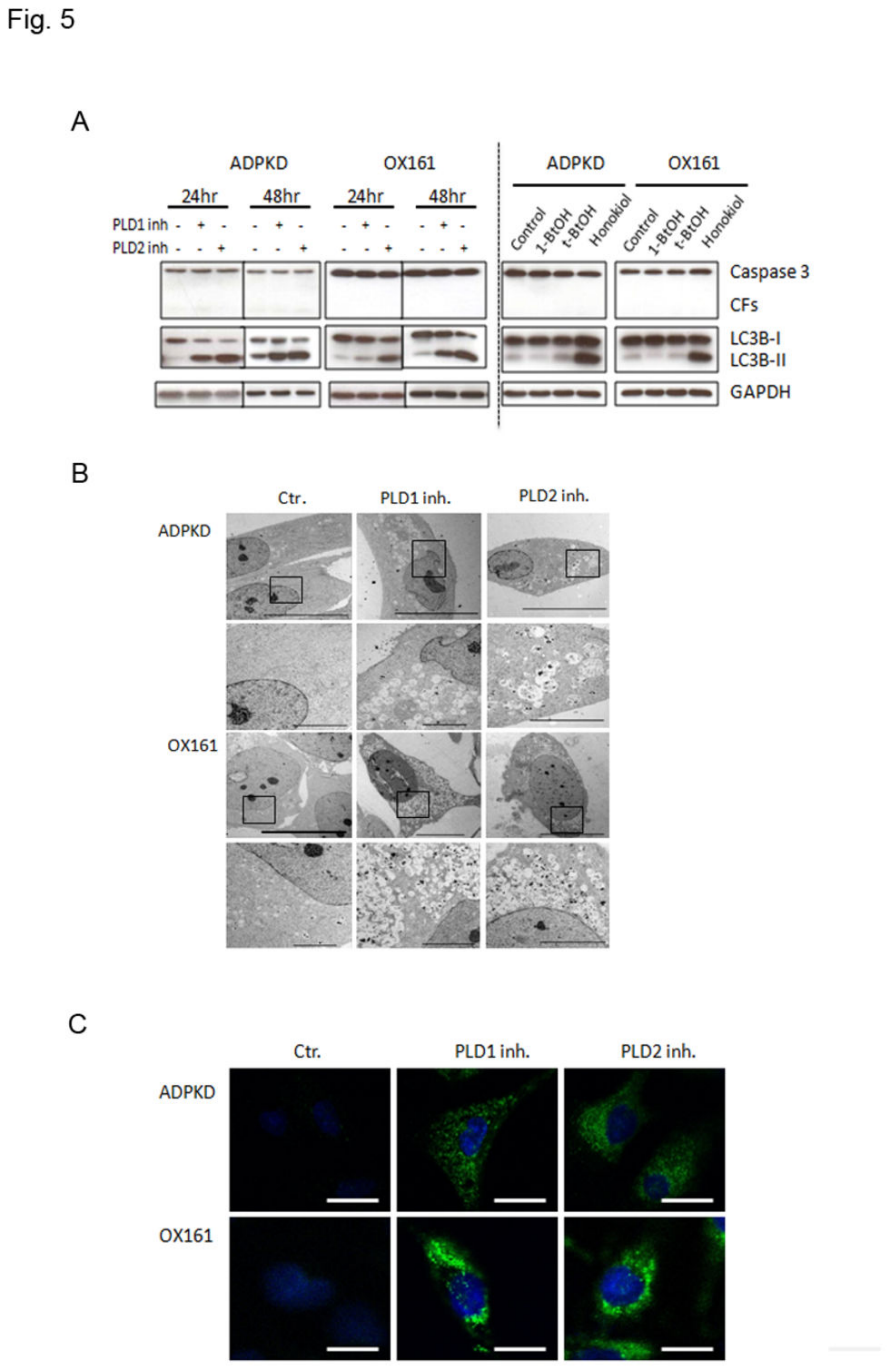
PLD inhibitors trigger autophagosome formation. (A) PLD inhibitors induced autophagy in a time-dependent way. Left panel: PKD cells were treated by either 10 µM PLD1 inhibitor or 20 µM PLD2 inhibitor for 24 h and 48 h. Right panel: PKD cells were plated for 24 h and then shifted to medium containing 0.5% serum. 1% 1-BtOH or 1% *tert*-BtOH was added for 2 h, 20 µM honokiol was added for 20 h. Western blots analyzing the expression of caspase-3, LC3B and GAPDH. (B) Transmission electron microscopy analysis of PLD1 and PLD2 inhibitor-treated HAK and OX161 cells (10 µM and 20 µM respectively, 48 h) showing intensive vacuolization and autophagosome-like vesicles. The lower panels show the magnification of the black frame area in the upper panels for each type of cell. The scale bar in the upper and lower panels represents 10 µm and 5 µm respectively. (C) Immunofluorescence microscopy analyzing autophagosome distribution after PLD1-/2- inhibitor (10 µM and 20 µM) treatment for 48 h. Cells were stained with anti-LC3B antibody. Scale bar represents 20 µm. Representative of three independent experiments.

mTOR is a master regulator of cell proliferation and modulates apoptosis and autophagy in many types of cells [4] [15]. To test whether PLD inhibitors affect downstream targets of mTOR, we examined the apoptosis and autophagy pathways after treatment with either PLD inhibitors, 1-BtOH, *tert*-BtOH or honokiol in ADPKD and OX161 cells. As shown in Figure **5A**, cleaved caspase-3, a marker of the apoptotic pathway, did not show an increased expression upon treatment. But immunoblotting analysis showed a drastic conversion of non-autophagic soluble LC3 (LC3-I) to autophagic LC3 (LC3-II) in response to PLD1- and PLD2- specific inhibitor and honokiol treatment in a time- and dose-dependent manner (Figure **5A**). One pathognomonic feature of autophagy is the ultrastructural evidence of autophagosomes. We examined PLD inhibitor-treated ADPKD and OX161 cells using transmission electron microscopy to visualize the induction of autophagy. Figure **5B** shows that after 48 h exposure to 10 µM PLD1- and 20 µM PLD2-inhibitors autophagosomes were abundantly present. To further examine the formation of autophagic vesicles, we performed immunofluorescence analysis for LC3. As expected, LC3 aggregated substantially in response to PLD inhibitors in both ADPKD and OX161 cells (Figure **5C**).

### Combinations of mTOR and PLD inhibitors have a synergistic effect on blocking the mTOR pathway in PKD cells

**Figure 6:**
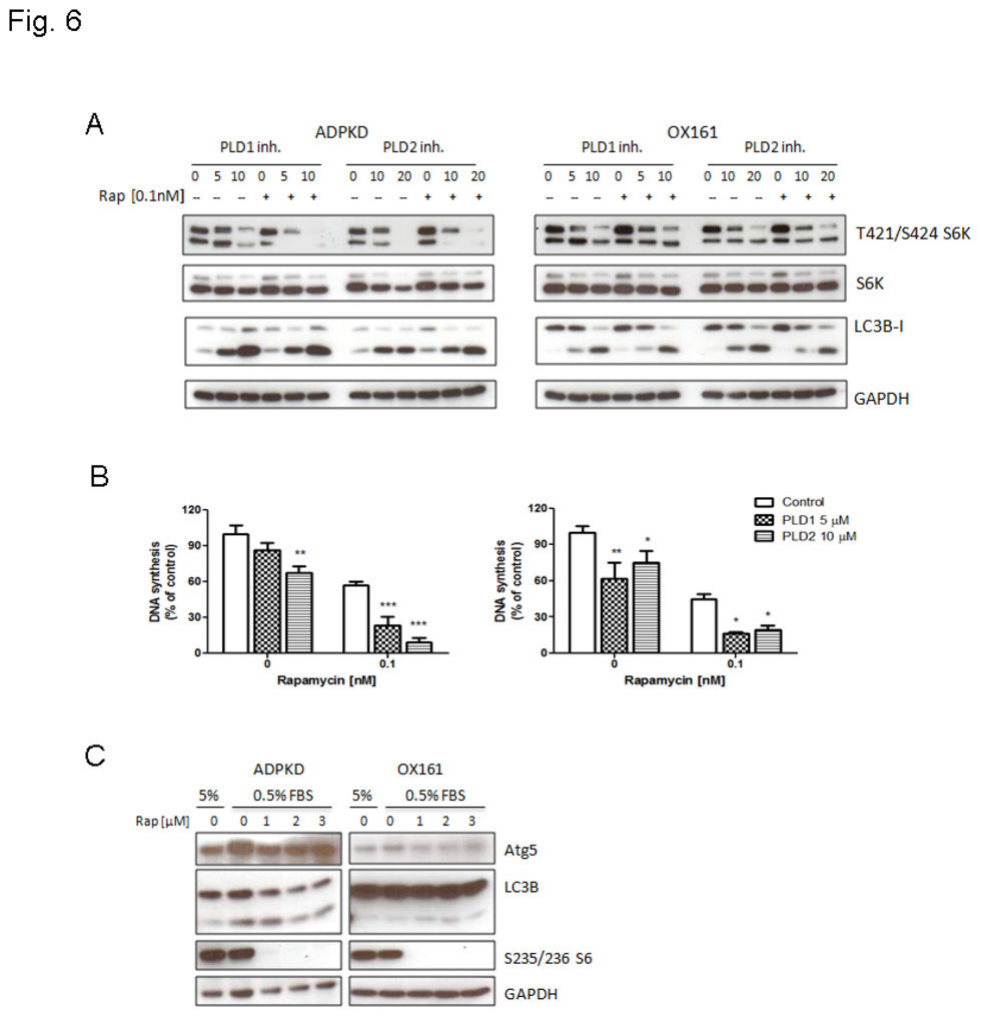
A combination of mTOR and PLD inhibitors has a synergistic effect on the mTOR pathway. (A) Combined mTOR and PLD1-/2-inhibitor treatment has a synergistic effect on blocking mTOR signaling in PKD cells. Western blots analyzing the expression of phospho-S6K, S6K, LC3B and GAPDH upon treatment with or without the indicated concentrations of PLD1-/2-inhibitor combined with 0.1 nM rapamycin for 48 h in PKD cells. (B) Combination treatment of mTOR and PLD inhibitors has a synergistic effect on blocking cell proliferation in PKD cell. BrdU assay determining DNA synthesis in human PKD cells upon treatment with or without the indicated concentration of PLD1-/2-inhibitor combined with 0.1 nM rapamycin for 48 h. (C) PKD cells display resistance to rapamycin-induced autophagy. Western blots analyzing the expression of Atg5, LC3B, phospho-S6 and GAPDH upon treatment with or without various concentration of rapamycin (1, 2, 3 µM) for 24 h under starvation conditions (culture medium containing 0.5% FBS). An experiment which is representative of three independent experiments is shown. Data are expressed as mean ± SD and were analyzed by one-way ANOVA. * p< 0.05, ** p< 0.01, *** p< 0.001.

The therapeutic targeting of mTOR in PKD has attracted attention in recent years due to a link between mTOR and survival signals in human PKD [28–30]. However, clinical trials with rapamycin and rapamycin analogues have been disappointing [31,32]. We hypothesized that the lack of efficacy could be due to elevated PLD activity that may had conferred resistance to rapamycin [33]. To examine whether blocking PLD activity leads to a decrease in the cellular resistance to rapamycin, we treated ADPKD and OX161 cells with a combination of low dose rapamycin and PLD inhibitors. The combinatorial treatment of rapamycin and PLD inhibitors exhibited more efficacy in blocking the phosphorylation of the S6 protein compared with PLD inhibitors treatment alone in ADPKD cells, but not in OX161 cells (Figure **6A**). Furthermore, we investigated whether the combination treatment had a synergistic effect in inhibiting PKD cell proliferation. As shown in Figure **6B**, neither 5 µM PLD1- nor 10 µM PLD2-inhibitor alone had a marked effect on cell proliferation. However, the combination treatment of 0.1 nM rapamycin with PLD1- or PLD2-inhibitors inhibited cell proliferation in both ADPKD and OX161 cells.

mTOR has been shown to negatively regulate autophagy upon nutrient limitation, which leads to suppressed mTOR pathway activity in many cell types. Therefore, we examined whether combining mTOR and PLD inhibitors had a synergistic effect on inducing autophagy. A Western blot analysis showed that LC-II conversion did not increase in response to the combination treatment compared with single agent treatment (Figure **6A**). We further assessed whether the low dose of rapamycin (0.1 nM) was not sufficient to induce autophagy in PKD cells. To this aim, we treated ADPKD and OX161 cells with a range of rapamycin concentrations for 24 h. Interestingly; even the highest concentration of rapamycin did not trigger autophagosome formation in PKD cells (Figure **6C**). However, the phosphorylation of the ribosomal S6 protein was completely blocked by the rapamycin treatment under these conditions (Figure **6C**) indicating successful blockage of the mTOR signaling pathway. Our results suggest that PKD cells are resistant to rapamycin-induced autophagy. Taken together, these results show that PLD inhibitors can reduce the resistance of PKD cells to rapamycin. Furthermore, combinations of mTOR and PLD inhibitors have a synergistic effect in reducing cell proliferation and mTOR pathway activation in PKD cells.

## Discussion

PKD1/PKD2 gene mutations causes aberrant signaling through the PC1-tuberin-mTOR complex leading to: 1) deregulated tuberin-mediated nutrient signaling [34]; 2) the vasopressin receptor 2-mediated accumulation of cyclic adenosine monophosphate (cAMP) [35]; and 3) abnormal activation of other pro-proliferative signaling pathways including Ras/Raf/ERK, cyclin-depedent kinases (Cdks), and JAK/STAT [36]. While there is a considerable amount of data supporting the hypothesis that the mTOR signaling pathway plays an important role in PKD, relatively little is known about the impact of PLD signaling on PKD [3,37]. Here we show for the first time that elevated PLD activity partly modulates the mTOR signaling pathway in PKD cells suggesting a biological role for PLD/PA in PKD progression.

In our study, we found mildly but significant elevated PLD activity in PKD cells compare with control cells. Blockade of PLD activity by either specific PLD inhibitors, honokiol or “alcohol trap” treatment lead to a reduction of PA levels and mTOR activity (Figures **2** and **4**, Figure **S1**) and in turn, profoundly decreased cell viability and proliferation and increased autophagosome formation. Firstly, PA is required for the association of mTORC1 with Raptor and mTORC2 with Rictor. PA activates mTOR complexes by interacting with the FKBP12-rapamycin binding (FRB) domain, the target of the mTOR inhibitor rapamycin [11]. Secondly, PA was found to specifically bind to and activate S6K, the downstream effector of mTORC1 by increasing phosphorylation in T389 and T421/S424 as well as S6K natural substrate protein S6 in 235/236 [38]. Furthermore, other proteins that are recruited or activated by the PLD product PA, for example PI4P5 kinase, PDK, Raf, Rac1 indicate that PLD/PA modulates survival signaling by mTOR but also by mTOR-independent signaling pathways [39]. To further elucidate the function of PLD/PA associated modulation of the mTOR pathway, we increased PA levels by adding exogenous PA to stimulate mTOR signaling. Phosphorylation of mTOR, p70S6K and Akt increased in a time-dependent manner, suggesting that PA activates both mTORC1 and mTORC2 pathways at multiple levels. Of note, exogenous PA reversed the anti-proliferative effect of PLD inhibitors, indicating that the effects of PLD inhibitors were governed not exclusively via mTOR signaling. These observations suggest that PLD/PA play an important role in modulating mTOR signaling pathway in PKD.

PLD activity in mammalian cells is mediated by two different isoforms, PLD1 and PLD2, which exhibit different regulation and subcellular locations [40,41]. The relative contribution of the two isoforms in mTOR pathway is not entirely clear but it appears that depending on the particular system, either PLD isoform can sustain mTOR pathway activation. In the present study, we used isoform-selective PLD inhibitors which have been reported to inhibit the respective isoforms with > 100-fold selectivity both in *in vitro* assays and in cells [26]. We confirmed the PLD isoform specific blockage of our applied inhibitors in human PKD cells. We observed that in PKD cells, mTOR activation was more sensitive to PLD1 than to PLD2 inhibition. The PLD1 inhibitor reduced more efficiently cell proliferation than the PLD2 inhibitor, which correlated with the sensitivity of the mTOR pathway activation to PLD isoform inhibition. The results have to be interpreted in the context that the dose-dependent effects of the PLD inhibitors only provide limited information regarding the role of these PLD isoforms in the mTOR pathway. It will therefore be important to investigate the subcellular localization of PLD isoforms and mTOR in PKD, as well as the effects of modulating the expression of different PLD isoforms on mTOR signaling in PKD cells in future studies. It should be noted that PLD inhibitors had anti-proliferative effects on both PKD and normal renal tubular epithelial cells.

Autophagy describes the process by which cytoplasmic materials including damaged or aged organelles and long-lived proteins reach the lysosomes for degradation and recycling by lysosomes [15]. Autophagy plays an important role in various aspects of cell physiology, especially cell survival during nutrient or energy limitation. However, autophagy can also trigger cell death and impair cellular functions in other contexts, underscoring its nature as a double-edged sword that can be either protective or injurious depending on the cellular environment, the nature and intensity of the stimulus, and the levels of autophagy [16,42]. There is so far only one published study reporting increased LC3-II conversion in homozygous Han: SPRD rat kidneys (Cy/Cy) at an advanced stage of PKD, indicating autophagy deregulation in PKD [18]. Aberrant signaling through the mTOR pathway is a common feature of PKD and there is a crosstalk between mTOR and autophagy in many types of cells [17,43]. In our study, mTOR inhibitors did not induce autophagy in PKD cells, even at high concentrations, suggesting that PKD cells are resistant to rapamycin-induced autophagy. Interestingly, we found that PLD inhibitors and honokiol treated PKD cells displayed autophagy suggesting that PLD-induced-autophagy bypassed the mTOR signaling pathway. In line with our findings, Takita T et al. reported that diacylglycerol kinase inhibitor reduced PA level induced autophagy in neuronal cells [44], whereas, Dall’ Armi et al. reported that PLD1 modulated autophagy via association with the endosomal system, partially re-localizing to the outer membrane of autophagosome-like structures [45]. Thus, PLD modulates autophagy in different manners, depending on cell type and subcellular localization: On one hand, PLD/PA interact with mTOR, PI3K-Ras/ERK signaling pathways, both regulate negatively autophagy. On the other hand, PLD1 has been shown to foster autophagy by co-localized with LC3 during starvation. Such a dual role has been previously described for Vps34, a lipid enzyme that is required for autophagy, yet also stimulates mTOR [46,47].

Rapamycin-based therapeutics effectively decreased cyst growth and preserved renal function in a variety of animal models for PKD [28,29,48]. However, conflicting results were obtained in clinical trials. Two clinical trials did not show a beneficial effect in ADPKD patients, both in early and more progressive disease stages [31,32]. One of the possible factors confounding the interpretation of these results could be the dose of mTOR inhibitors used. The levels of rapamycin tolerated in humans are lower than in mice [28]. Based on our results, elevated activation of PLD may mitigate the effect of rapamycin on human PKD cells. Indeed, combining mTOR and PLD inhibitors enhanced the rapamycin-sensitivity of PKD cells. Therefore, combination therapies that include rapamycin and strategies that suppress PLD activity could be used to target mTOR signaling in PKD.

**Figure 7:**
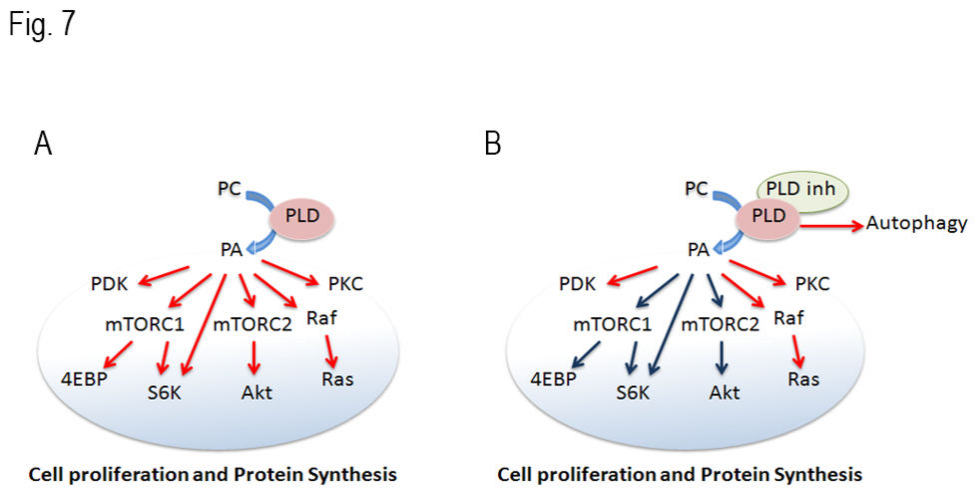
PLD and mTOR pathway in PKD. (A) Elevated PLD activity stimulates the mTORC1, mTORC2 and other relative pathways and promotes cell proliferation in PKD cells. (B) Inhibition of PLD reduces phosphorylation of downstream targets of mTORC1 and mTORC2, and induces autophagy in PKD cells.

In summary, our data shows that elevated PLD activity in PKD cells. mTOR signaling pathway was partly modulated in a PLD/PA-dependent way (Figure **7**). Targeting PLD blocked cell proliferation, decreased mTOR signaling and induced autophagy formation. Combination of mTOR and PLD inhibitors has a synergistic effect on retarding cell proliferation and blocking mTOR pathway. Targeting PLD may provide a new potential therapeutic approach for PKD.

## Supporting Information

Figure S1 **Honokiol impaired cell growth and blocked mTOR signaling in Cy/**+** cells.** Effect of honokiol on (A) cell viability and DNA synthesis determined by MTS and BrdU. 48 h after treatment initiation. (B) Honokiol affected PLD/PA and mTOR signaling in a dose-dependent way. Western blots analyzing the expression of phospho-PLD1, PLD1, phospho-PLD2, PLD2, phospho-Akt, Akt, phospho-S6K, S6K, phospho-S6 and S6 either upon treatment with the indicated concentration of PLD1-/2- inhibitor for 48 h or without treatment. Blots are representative of three independent experiments. Data are expressed as mean ± SD and were analyzed by one-way ANOVA. * p< 0.05, ** p< 0.01.
(TIF)
