# Supplementary figures and images for: The Role of Phospholipase D in Modulating the MTOR Signaling Pathway in Polycystic Kidney Disease
Source: PLoS One. 2013 Aug 23;8(8):e73173. doi: 10.1371/journal.pone.0073173 (PMC3751888; doi:10.1371/journal.pone.0073173)

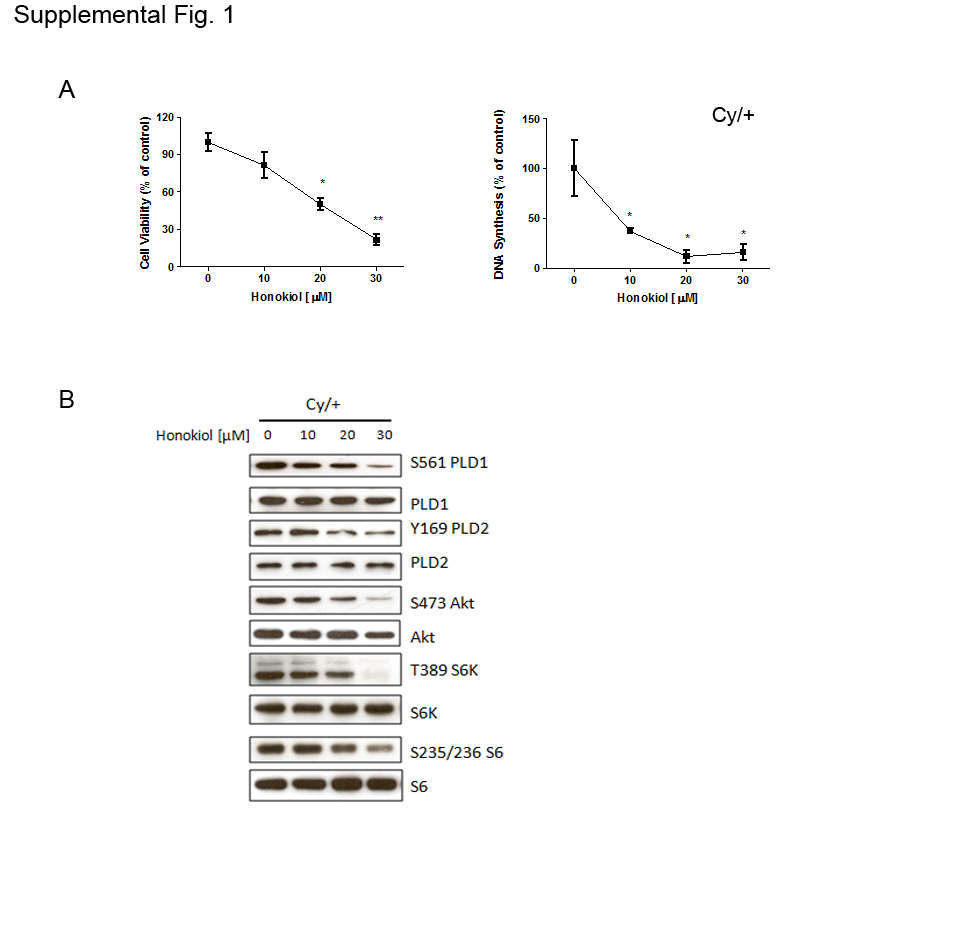

Supplement: Figure S1 — Honokiol impaired cell growth and blocked mTOR signaling in Cy/+ cells. Effect of honokiol on (A) cell viability and DNA synthesis determined by MTS and BrdU. 48 h after treatment initiation. (B) Honokiol affected PLD/PA and mTOR signaling in a dose-dependent way. Western blots analyzing the expression of phospho-PLD1, PLD1, phospho-PLD2, PLD2, phospho-Akt, Akt, phospho-S6K, S6K, phospho-S6 and S6 either upon treatment with the indicated concentration of PLD1-/2- inhibitor for 48 h or without treatment. Blots are representative of three independent experiments. Data are expressed as mean ± SD and were analyzed by one-way ANOVA. * p< 0.05, ** p< 0.01. (TIF) [file pone.0073173.s001.tif]
